# Impact of Pressure Variability and Comorbidities on PAP Therapy Compliance and Adherence in Obstructive Sleep Apnea

**DOI:** 10.3390/life16010048

**Published:** 2025-12-27

**Authors:** Ioana Munteanu, George Alexandru Diaconu, Constantin Gheorghevici, Nicolae Feraru, Beatrice Burdusel, Catalin Constantin Coca, Florin Dumitru Mihaltan, Beatrice Mahler, Sergiu Ioachim Chirila, Valeria Herdea

**Affiliations:** 1 Marius Nasta Institute of Pneumology, 050159 Bucharest, Romania; ioana.munteanu2015@yahoo.ro (I.M.); mihaltan@starnets.ro (F.D.M.);; 2 Department of Physiology, Faculty of Medicine, “Titu Maiorescu” University, 040441 Bucharest, Romania; 3 Department of Pneumology, Faculty of Medicine, “Carol Davila” University of Medicine and Pharmacy, 050474 Bucharest, Romania; 4 Department of Medical Informatics and Biostatistics, Faculty of Medicine, “Ovidius” University, 900470 Constanta, Romania; 5 Romanian Association for Pediatric Education in Family Medicine (AREPMF), 021507 Bucharest, Romania; valeriaherdea@yahoo.com

**Keywords:** CPAP adherence, obstructive sleep apnea (OSA), auto-PAP pressure variability, comorbidities, sleep-disordered breathing

## Abstract

Obstructive sleep apnea syndrome (OSAS) is a common disorder with established cardiovascular and metabolic risks. Positive airway pressure (PAP) therapy remains the standard of care; however, its long-term effectiveness is often limited by poor compliance and adherence. This study sought to explore clinical and device-related factors influencing PAP use, with emphasis on pressure variability in Auto-PAP users and comorbidities such as COPD. We performed a retrospective analysis of 359 patients with OSAS who were treated with CPAP, Auto-PAP, or BiPAP devices at the Marius Nasta Institute of Pneumology between January 2022 and July 2024. Compliance was measured as the proportion of days the device was used, whereas adherence was estimated through average nightly hours of use. Patient data were stratified by demographic, clinical, and device-related characteristics. Statistical testing included Chi-square, Wilcoxon rank-sum, and correlation analyses. Demographics did not significantly differ between compliant and non-compliant groups. Notably, Auto-PAP users with greater pressure variability (>10 cm H_2_O) had significantly lower compliance (*p* = 0.001). Nasal mask preference was also associated with poorer compliance (*p* = 0.030). Multivariate models further revealed that atrial fibrillation reduced the likelihood of good adherence (OR = 0.319, 95% CI 0.137–0.746). These results highlight the importance of monitoring pressure variability, device type, and comorbidities to personalize PAP therapy and improve long-term outcomes.

## 1. Introduction

Obstructive sleep apnea syndrome (OSAS) is the most common form of sleep-disordered breathing. The consequences of OSAS are well-documented and include nocturnal hypoxemia, excessive daytime sleepiness, sleep fragmentation, impaired cognitive function, and an increased cardiovascular and metabolic burden [1]. Cardiovascular and metabolic complications have the potential to lead to pathologies such as heart failure, stroke, or diabetes [2].

Starting in 2022, Romania introduced the possibility of reimbursement of positive airway pressure devices by CNAS (National Health Insurance House), thereby facilitating the diagnosis and treatment of OSAS. Patients are required to attend follow-up visits every three months and bring the report generated by the PAP device to assess treatment adherence and compliance in order to maintain eligibility for reimbursed therapy.

The specialist analyzes the usage report of the device and issues medical prescriptions for the PAP device if the patient’s adherence rate (the number of days with more than 4 h of usage in relation to the total number of days on which the usage report was issued) is greater than 70% ([3], p. 46). The patient takes the prescriptions to the headquarters of the National Health Insurance House and receives a free voucher for a standard period of 3 months. Patients who hold a certificate of severe disability or have an attendant receive a prescription for a PAP device for one year.

In 2024, we published the first guidelines for the management of adult patients with sleep-disordered breathing in Romania, which contain key recommendations for the follow-up of patients with sleep apnea. In the national guidelines for the management of adult patients with sleep-disordered breathing during sleep, endorsed by the Ministry of Health and various experts in the field, the importance of adherence and compliance of patients using PAP devices is specified, and the way in which patients with sleep apnea syndrome should be diagnosed, treated, and followed up is standardized. The follow-up of patients with sleep-disordered breathing is, according to the national guidelines, done one month after the initiation of PAP therapy, then at three months, six months, and annually thereafter. However, the National Health Insurance House requires patients to visit their doctor every three months, where the sleep expert analyzes the report generated by the device provider.

Discontinuation of PAP therapy without medical guidance typically results in the recurrence of symptoms, often prompting patients to seek re-evaluation and re-initiation of diagnostic and therapeutic procedures (nocturnal cardiorespiratory polygraphy or polysomnography), titration (automatic or manual), and treatment (CPAP, Auto-PAP, or BiPAP). This entails high costs and significant material and human resources.

The CPAP device, or continuous positive airway pressure, delivers a single pressure throughout its use, and therefore throughout the breathing cycle.

BiPAP (Bi-Level positive airway pressure), or BPAP, delivers a higher pressure during inspiration (IPAP) and a lower pressure during expiration (EPAP), generating a ventilatory effect.

The Auto-PAP, Auto-CPAP, and APAP, i.e., automated (continuous) positive airway pressure, automatically adjust the delivered pressure according to the patient’s needs, within a range set by the medical staff [3].

Our paper aims to analyze the data of patients who have already used positive airway pressure devices (thus having relatively good usage statistics if they accessed them via the reimbursement system) or received one for sleep apnea syndrome during 1 January 2022–1 July 2024 at the Marius Nasta Institute of Pneumology, Bucharest, Romania. Therefore, this study aims to identify clinical characteristics associated with non-adherence or non-compliance to PAP therapy and to explore potential strategies to improve treatment adherence and compliance. Additionally, we aim to evaluate the impact of PAP pressure settings on patient adherence and compliance, with a specific focus on the effects of high-pressure variability in Auto-PAP users; to assess the role of comorbidities in therapy adherence and compliance by identifying which clinical conditions are associated with non-compliance; and to analyze the influence of demographic and geographic factors on PAP adherence and compliance by comparing adherence rates between urban and rural populations, exploring potential barriers to compliance, and determining whether different mask interfaces (nasal vs. oronasal) contribute to variations in patient compliance.

## 2. Materials and Methods

This is a retrospective-descriptive study that included 359 patients who were evaluated in the Marius Nasta Institute of Pneumology, Bucharest, Romania, between 1 January 2022 and 1 July 2024. We included and analyzed all entries with sleep apnea-related diagnostic codes from the hospital information system. Anamnesis, clinical course, and objective examinations (including interpretation of device-generated reports) were conducted by somnology-accredited medical doctors employed at the Marius Nasta Institute of Pneumology. Device allocation and pressure settings were done by a sleep specialist, either from the Institute or from another clinic.

We gathered a total of 18795 entries (day hospitalizations), which were attributed a somnology payment service (cardiovascular polygraphy, polysomnography, adjustment of device settings, prescribing documents for gratuity of the positive pressure device). We identified 654 unique patients who met the initial inclusion criteria (identification data, positive diagnosis of sleep apnea syndrome, (meaning an apnea--hypopnea index (AHI) of at least 5/h), age above 18), of which 295 patients did not return to the hospital for follow-up at the first 3-month follow-up visit from the initial consultation and were, therefore, excluded. Being a strictly computerized input study, the exclusion criteria were failure to prescribe a positive airway pressure delivery device and failure to attend the first follow-up after initiation of CPAP treatment.

All other entries in the computer system represented patients over 18 years of age who signed informed consent.

Device usage data were extracted from the memory card by the supplier company, which generates a compliance or usage report. Patients present this report to the treating physician during follow-up visits for interpretation and validation. Selected essential data from this report (adherence, compliance, residual apnea-hypopnea index (AHI)) were then transcribed into the hospital’s computer system, in the patient’s chart.

The initial follow-up was done at 1–3 months. Subsequent follow-up intervals were influenced by factors such as device ownership status (purchase or rental), patient availability, and technical or clinical issues encountered during the initial three-month period.

We analyzed data such as the severity of sleep apnea syndrome; the type of device used (CPAP/Auto-CPAP/BPAP); cardiovascular, respiratory, and metabolic comorbidities; device pressure and pressure support (for NIV-BPAP users); the type of mask used; compliance; adherence; and the residual apnea-hypopnea Index. The resulting database was introduced and analyzed in R [4]. Descriptive statistics (mean, standard deviation, frequency, and percentages) were used to summarize patient characteristics, CPAP adherence and compliance, and comorbidities. All *p*-values were two-sided, and a *p*-value < 0.05 was considered statistically significant. Comparative analyses were performed using the Chi-square test (or Fisher’s exact test) for categorical variables and the Wilcoxon rank-sum test for continuous variables. Binary logistic regression was used to evaluate the individual effects of medical conditions and demographics on adherence and compliance. Correlation analyses (Pearson or Spearman, as appropriate) were conducted to assess relationships between adherence and compliance and factors such as pressure variability and comorbidities.

**Compliance** was defined as the proportion of days during which the patient used the positive airway pressure (PAP) device, irrespective of the duration of use, relative to the total number of days covered by the utilization report.

**Adherence** was defined as the mean number of hours the patient used the positive airway pressure (PAP) device per night, as recorded in the utilization report. This continuous, hour-based metric provides a direct and objective measure of nightly device usage.

**Pressure variability** was calculated as the difference between the maximum and minimum delivered pressures recorded in the device summary report (Pmax − Pmin). While more granular metrics (such as the 90th or 95th percentile pressure) provide detailed physiological insights, the summary nature of the retrospective database limited our analysis to the absolute pressure range. This metric serves as a proxy for the pressure instability experienced by the patient.

**Adherence rate** is defined as the percentage of days with device usage exceeding four hours per night over the typical three-month reporting period.

While compliance data (proportion of days used) was available for the entire cohort (n = 359), data regarding specific adherence (average nightly duration of use) was available for 211 patients (58.8%). The missing data for the remaining 148 patients (41.2%) was solely due to administrative reasons: the specific hour-based metric was not transcribed from the device report into the hospital’s electronic database by the medical staff during the consultation. As these omissions were clerical and unrelated to patient characteristics or usage patterns, the data were considered missing completely at random (MCAR). The study cohort consisted of 359 unique patients who presented further inclusion criteria (users of CPAP/AutoPAP/BiPAP machines and a minimum of 1 follow-up consult).

Given that the primary objective of this study was to assess adherence and compliance to positive airway pressure (PAP) therapy, only patients who attended follow-up consultations required to maintain eligibility for reimbursed PAP devices were included in the analysis. This implies an overall adherence rate of at least 70%. For the purpose of this study, patients with a mean nightly device usage of less than 5 h were classified as having poor adherence, while those with a mean nightly usage of 5 h or more were considered to have good adherence. The number of patients with a mean nightly usage below 4 h was negligible and did not significantly influence the results.

This study was approved by the Scientific Research Ethics Committee of the Marius Nasta Institute of Pneumology in Bucharest, Romania, under the number 14175/11 July 2024.

## 3. Results

**Patient characteristics**

A total of 359 patients were included in this study. All of them were, at the moment of data collection, positive-pressure device users. The mean age was 59.76 years. Approximately three-quarters of them lived in Bucharest-Ilfov County (76.7%), 19.17% lived in provinces, and 4.13% did not specify where they lived.

### 3.1. Demographic and Clinical Characteristics vs. Compliance

Among the 359 patients, no statistically significant differences were observed in demographic characteristics (sex, age, smoking status, or living environment) between the good and poor compliance groups (Table 1). However, a statistically significant lower prevalence of arterial hypertension was found in the poor compliance group (54%) compared to the good compliance group (74%) (*p* = 0.025) (Table 2), potentially linked to the trend toward slightly younger age among those with lower compliance, though the age difference was not statistically significant (*p* = 0.094). Notably, patients with nasal septum deviation were more prevalent in the good compliance group (25%) versus 7.7% in the poor compliance group (*p* = 0.049).

### 3.2. Device Type and Sleep Study Results vs. Compliance

No significant differences in the apnea-hypopnea index (AHI), oxygen desaturation index (ODI), or oxygenation parameters during polygraphy were observed between the compliance groups. However, Auto-PAP users tended to be overrepresented in the poor compliance group (81% vs. 63%, *p* = 0.13) (Table 3), suggesting a possible trend, although it was not statistically significant. In contrast, mask type showed a significant association: nasal mask use was higher among poor compliers (50%) compared to 27% among good compliers (*p* = 0.030).

### 3.3. Pressure Settings vs. Compliance

**Figure 1:**
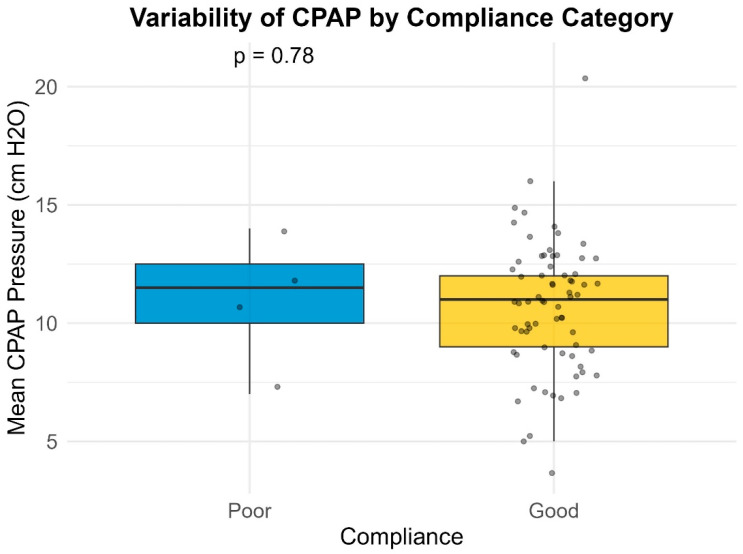
Variability of CPAP by compliance category.

**Figure 2:**
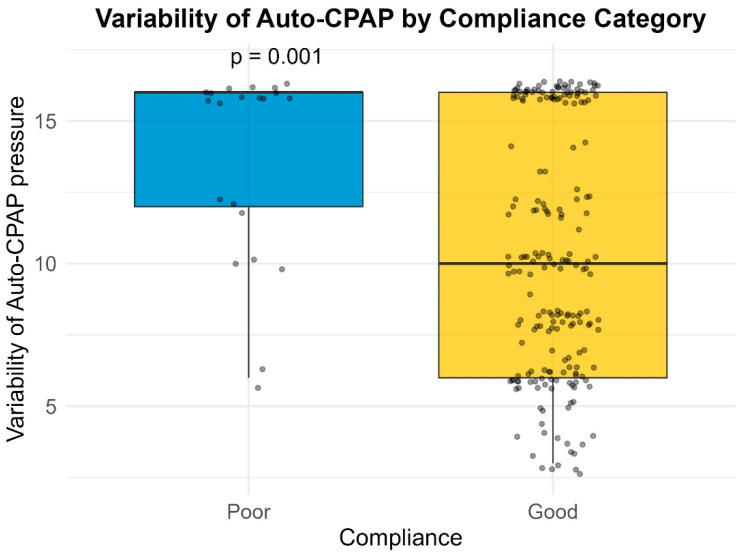
Variability of Auto-PAP by compliance category.

**Figure 3:**
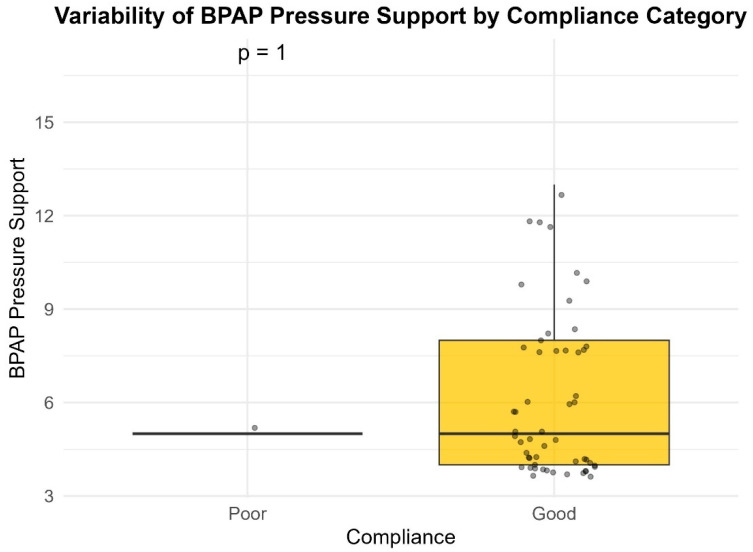
Variability of BiPAP pressure support by compliance category.

**Figure 4:**
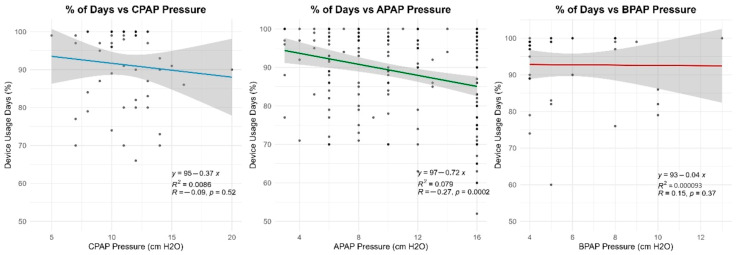
Compliance trends in relation to the type of device used.

Median pressure settings did not significantly differ between groups for CPAP or BiPAP users. However, patients with poor compliance exhibited significantly higher variability in Auto-PAP pressures (median variability = 16.0 cm H_2_O) compared to good compliance users (median variability = 10.0 cm H_2_O; *p* = 0.001) (Table 4) (Figure 1, Figure 2, Figure 3 and Figure 4). This finding suggests that greater pressure variability (CPAPmax − CPAPmin > 10 cm H_2_O) may negatively impact device usage consistency.

### 3.4. Demographics vs. Adherence

In the adherence analysis (n = 211), demographic variables including sex, age, and smoking status did not differ significantly between poor and good adherence groups (Table 5). Urban residents were more likely to be adherent (88%) than rural residents (82%), though this difference was not statistically significant (*p* = 0.24).

### 3.5. Comorbidities and Anatomical Features vs. Adherence

Among clinical characteristics, the presence of atrial fibrillation was significantly higher in the poor adherence group (27%) compared to the good adherence group (14%) (*p* = 0.018), indicating a possible association. Nasal septum deviation was more common in adherent patients (34% vs. 20%, *p* = 0.023), while no differences were seen in obesity status, diabetes, or COPD prevalence (Table 6).

### 3.6. Sleep Parameters and Device Type vs. Adherence

No significant differences were observed in AHI or minimum oxygen saturation between adherence groups. However, a higher ODI was noted in the poor adherence group (median 52 vs. 44, *p* = 0.026). Device type was significantly associated with adherence: Auto-PAP users were more frequently found among poor adherents (73% vs. 56%; *p* = 0.027), while CPAP users were more common in the good adherence group (27% vs. 16%) (Table 7).

### 3.7. Pressure Settings vs. Adherence

**Figure 5:**
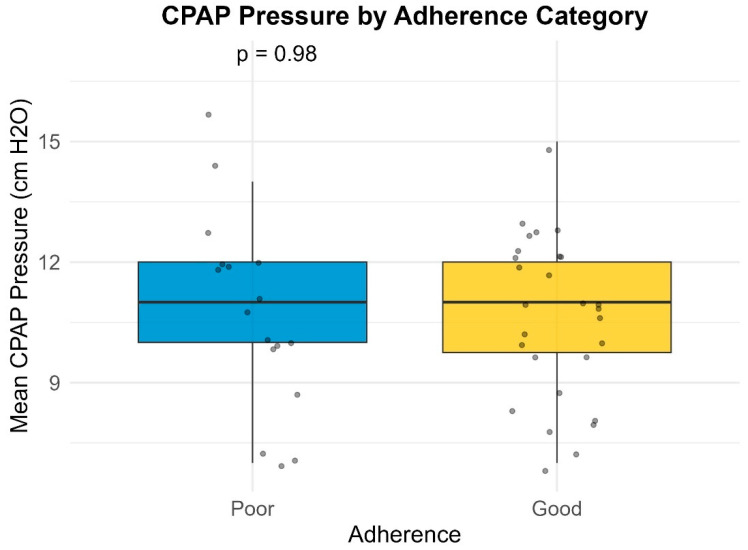
CPAP pressure by adherence category.

**Figure 6:**
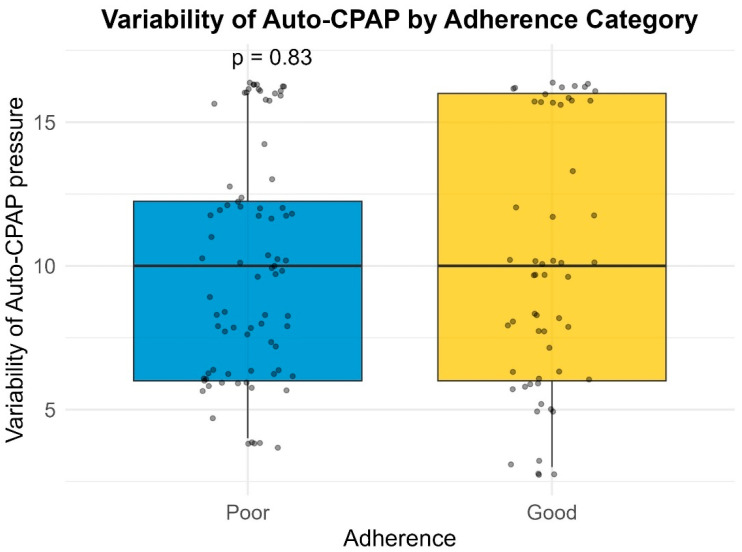
Variability of Auto-PAP by adherence category.

**Figure 7:**
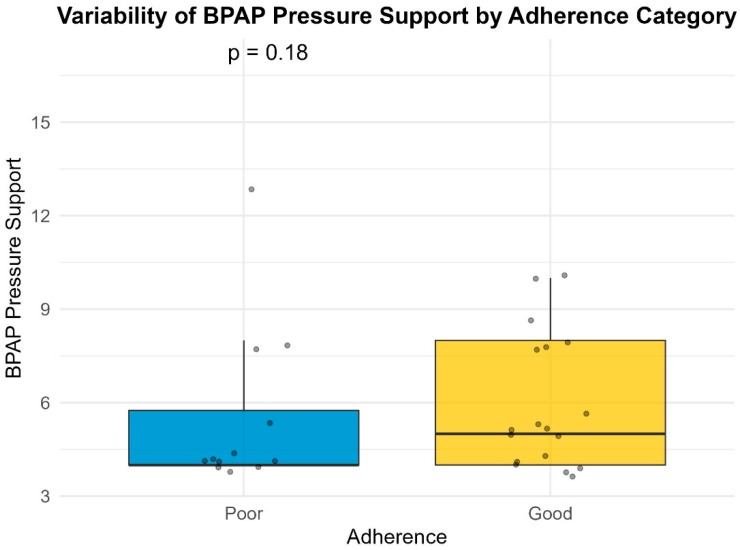
Variability of BiPAP pressure support by adherence category.

**Figure 8:**
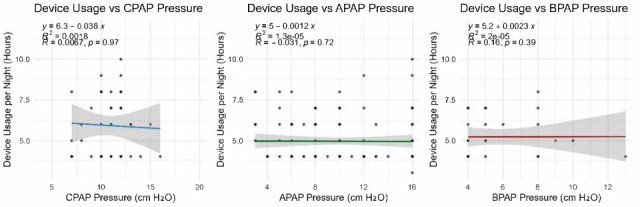
Adherence trends in relation to the type of device used.

Pressure settings and pressure variability did not significantly differ between adherence groups. This suggests that, unlike compliance, adherence (when measured by hours of usage) may not be directly influenced by pressure values or Auto-PAP variability (Table 8, Figure 5, Figure 6, Figure 7 and Figure 8). It should be noted that many of the data related to the number of hours of use were missing from the medical records.

Correlation analyses between pressure settings and adherence (measured in usage hours per night) revealed no clinically relevant associations. Correlation coefficients remained below 0.2 for all pressure modalities, indicating that pressure level alone does not determine nightly usage duration.

### 3.8. Multivariate Analysis

We conducted a binomial regression to evaluate the effects of sex, age, smoking status, and medical conditions on the likelihood of good compliance. The logistic regression model was not statistically significant, χ^2^(16) = 21.56, *p* = 0.158. The model explained 21.8% (Nagelkerke R^2^) of the variance in compliance and correctly classified 91.1% of the cases. The Area Under the ROC curve was 0.797 (95% CI 0.696–0.899), indicating acceptable discrimination. Arterial hypertension was identified as a potential factor associated with compliance in a statistically significant way, with a *p* value of 0.017, indicating an OR of 5.233 with 95% CI 1.345–20.357 (Table 9).

We also conducted a binomial regression to evaluate the effects of sex, age, smoking status, and medical conditions on the likelihood of good adherence. The logistic regression model was statistically significant χ^2^(16) = 30.330, *p* = 0.016. The model explained 23.3% (Nagelkerke R^2^) of the variance in adherence and correctly classified 53.8% of the cases. The Area Under ROC curve was 0.755 (95% CI 0.679–0.832, *p* < 0.001), indicating acceptable discrimination. Of the predictor variables included in the model, the presence of atrial fibrillation, nasal septum deviation, and type of device were statistically significant (as seen in Table 10).

Patients with atrial fibrillation had significantly lower odds of having good adherence (OR = 0.245, 95% CI 0.88–0.684), patients with nasal septum deviation had significantly higher odds of having good adherence (OR = 2.513, 95% CI 1.047–6.029), and patients with APAP (variable pressure) had statistically significantly higher odds of having good adherence compared to other types of devices, with an OR of 2.826 (95% CI 1.129–7.071).

## 4. Discussion

The National Health Insurance House (CNAS) has established specific eligibility criteria for the continuation of reimbursed treatment for obstructive sleep apnea syndrome (OSAS). These include proof of insured status (e.g., evidence of social health insurance contributions or student status), a nocturnal cardiorespiratory polygraphy demonstrating moderate or severe OSAS, or mild OSAS with clinically significant symptoms, documented device usage of at least four hours per night on more than 70% of monitored days (indicative of good adherence) and regular follow-up consultations with a sleep specialist every three months [4,5].

As we mentioned in the Methods section, the population included in this study represents patients who were able to access the national compensation program for PAP devices by meeting the legal requirements and having good initial compliance to justify the continuation of the reimbursement. This is reflected in our study by the high percentage of patients with good compliance and adherence, greater than that in other reports in the literature. The 359 patients included in this study represent those who were prescribed a positive airway pressure device and underwent at least one evaluation, and therefore initially met the legal conditions for receiving a gratuitous device. Although all patients initially met the eligibility criteria established by the National Health Insurance House, a subset later demonstrated suboptimal treatment outcomes. This subgroup represents the non-compliant or non-adherent population analyzed in this study. Therefore, our study focuses on secondary non-adherence (discontinuation after successful initiation) rather than primary non-adherence. This specific population is highly relevant for analyzing the cost-efficiency of the reimbursement system.

CNAS provided us with data related to the reimbursement of respiratory devices to patients in 2023, as follows: 16,565 patients rented Auto-PAP devices (26,945 coupons deducted), 1922 patients rented CPAP devices (2529 coupons deducted), and 1488 patients rented BiPAP devices (2341 coupons deducted) [6].

The number of patients who received a single voucher in 2023 (no prescription/indication to continue therapy or no longer wished to continue therapy) was as follows: 594 APAP patients (3.58%), 82 CPAP patients (4.26%), and 78 BiPAP patients (5.24%) [7,8].

BiPAP therapy, in its various modes (Spontaneous, Spontaneous/Timed with AVAPS on or off), is recommended for patients who have alveolar hypoventilation (such as Obesity-Hypoventilation Syndrome or hypercapnic COPD who also have OSA) or for patients with OSA who require high-pressure values or cannot tolerate CPAP [4]. This is the reason that BiPAP devices were prescribed in approximately 15% of the patients included.

Adherence and compliance with positive airway pressure devices are important tools to monitor patient outcomes and reduce costs related to this pathology. The aforementioned complications, such as cardiovascular and metabolic pathologies, can be avoided by PAP treatment, thus further reducing costs [7,8].

Although the terms adherence and compliance are often used interchangeably in the scientific literature, the Romanian National Guidelines for the Management of Sleep-Disordered Breathing in Adults explicitly differentiate between these concepts. Furthermore, the utilization reports generated by the software used to extract data from PAP device memory cards report both parameters separately, according to the definitions provided in the “Methods” section of this manuscript [4].

An et al. published a 36-month study in 2023, in which they concluded that patients with high adherence to SAS treatment had approximately 50% lower costs than those with low adherence [8]. We do not yet have access to data regarding treatment costs for sleep apnea syndrome.

Although the data made available by CNAS for the year 2023 does not identify Auto-PAP patients as the most likely to drop out of therapy, our data “in the field” highlights a 12.62% poor adherence rate among those on APAP treatment [5]. Furthermore, only 15% of patients in the study population had a diagnosis of COPD, and among those with poor adherence, the percentage was 25.7%.

The physician plays a critical role in promoting treatment, adherence, and compliance. Key aspects, such as addressing therapy-related side effects, ensuring proper mask and harness fitting, and engaging in patient-centered discussions during follow-up visits, are essential for optimizing compliance. The patient’s wishes, as well as any changes in sleep behavior, should be taken into account, and any related problems should be resolved [7,9]. A modest improvement in adherence and compliance was observed over the course of this study, particularly following multiple follow-up consultations.

Notably, patients residing outside the Bucharest-Ilfov region demonstrated lower compliance, as measured by average nightly device usage, compared to those living within the capital area. Although not statistically significant, this could imply that more regular check-ups or being close to a major hospital can play a role in improving compliance and adherence.

A study published in 2022 by Rosa et al. concluded that the patient’s partner can play a significant role in increasing adherence and compliance to treatment. It also mentions the existence of a “sleep nurse” and her role as a “bridge” between the sleep physician and the patient/couple [10].

A specialized training program for nurses introducing the role of the “sleep nurse” has recently been initiated in Romania. Their role is, as of now, limited to setting up components in polysomnographies and nocturnal polygraphies, taking care of the patient during the investigation, and generating sleep reports. In the future, their role could extend to following up with the patient, fine-tuning the device, and detecting broken or overused masks and other components.

Many of the patients (27.7%) included in our study had a large variability in their Auto-PAP pressures (4–20 cm H_2_O). Our results indicate a slight inverse proportional relationship between the variability in Auto-PAP pressures (such as CPAPmin = 4 cm H_2_O and CPAPmax = 20 cm H_2_O) and compliance or adherence to treatment. These findings suggest that adequate pressure titration may improve patient tolerance. A discussion among somnologists about the superiority of manual pressure titration is thus warranted.

Compliance and adherence to positive airway pressure treatment may also be influenced by other factors that we did not analyze in this study. For example, in a previously published study, we analyzed sleep quality in post-COVID patients and concluded that more than half of the included patients reported unsatisfactory sleep quality (PSQI > 5) [11].

Similarly, an article published by Zhang, L. et al. showed that 90% of the patients included in their study reported poorer sleep after being infected with SARS-CoV-2. Poor sleep quality led to longer hospitalizations and immune system dysregulation [12].

Multiple sources in the literature indicate that non-compliance with positive airway pressure therapy is related to the “adverse effects” of positive airway pressure therapy, such as air leaks, mask mismatch, ophthalmic discomfort, and device noise [12,13,14]. Device interface preferences did not appear to significantly impact adherence. While 53.4% of patients used an oronasal mask and 17.4% a nasal mask, no clear association between mask type and adherence was found. The relatively high proportion of nasal mask usage is likely attributable to their substantially lower cost, which is approximately 50% of that of oronasal masks. Currently, in Romania, PAP device masks are not reimbursed by the National Health Insurance House, but some providers offer them as a bonus when signing a rental contract for a PAP device. However, missing data on subjective mask discomfort limits the ability to draw firm conclusions. Surprisingly, our multivariate analysis identified nasal septum deviation as a predictor of better adherence (OR = 2.513). Although nasal pathology is traditionally viewed as a barrier to PAP tolerance due to increased airflow resistance, we hypothesize that these patients may experience a more dramatic subjective improvement in sleep quality. The positive pressure delivered by the device acts as a ‘pneumatic splint’, overcoming the fixed anatomical resistance of the deviated septum. Consequently, these patients might perceive a significant reduction in the work of breathing during sleep compared to their baseline, potentially driving higher motivation and adherence compared to patients whose obstruction is solely pharyngeal. Future research should consider patient-reported outcomes, such as comfort and side effects, to refine mask selection criteria for improved long-term compliance and adherence.

Broström et al. postulated in their study that not understanding side effects may lead to poor therapy adherence. Healthcare personnel providing education should prioritize identifying individual learning needs and recognizing barriers related to the disease and psychosocial factors. The authors also mentioned that it is important to discuss patients’ experiences of suffering and external pressures that might negatively affect treatment adherence. Understanding barriers to learning in patients with obstructive sleep apnea syndrome (OSAS) is crucial since long-term sleep deprivation can lead to mood and cognitive issues, impacting learning capacities. A varied didactical approach, utilizing small groups and different methods (practical, verbal, written, video), may enhance learning outcomes. Behavioral theories should be applied to boost self-determination and motivation. Emphasizing the positive effects of treatment can encourage informed choices, while later educational stages should address risk factors and promote dietary changes for overall health. Setting realistic goals for patient involvement in their care and evaluating learning outcomes at every educational stage is essential [15].

Another study by Broström et al. highlighted the significant impact of CPAP therapy on improving sleep quality and reducing symptoms of obstructive sleep apnea (OSA). The authors found that although CPAP usage led to notable improvements in daytime sleepiness, cognitive function, and overall quality of life, adherence to therapy remained a critical challenge. Many patients reported side effects such as nasal congestion, dry mouth, and mask discomfort, which contributed to lower compliance rates. The findings underscore the importance of addressing these barriers through patient education, proper device fitting, and ongoing support to enhance long-term adherence and maximize the therapeutic benefits of CPAP for OSA patients. In our study, we did not have access to data regarding mask discomfort or any other type of subjective complaint regarding the usage of the PAP device [16].

Patient compliance with positive airway pressure therapy has become a subject of study since its widespread use. A study published in *Chest* in 1995 analyzed the most common adverse effects reported by patients. The study examined the effectiveness of CPAP therapy in managing obstructive sleep apnea (OSA) and explored factors influencing patient compliance and adherence. The authors found that CPAP significantly improved respiratory outcomes, reduced apnea-hypopnea index (AHI) scores, and alleviated symptoms such as daytime sleepiness. However, adherence to CPAP therapy was suboptimal, with many patients discontinuing use due to side effects like nasal dryness, mask discomfort, and claustrophobia. The study emphasized the need for tailored patient education, early intervention to address side effects, and regular follow-ups to improve long-term adherence. These findings highlight the dual challenges of ensuring therapeutic efficacy while addressing practical barriers to consistent CPAP use [17].

The study “Obstructive Sleep Apnea Syndrome Comorbidity Phenotypes in Primary Health Care Patients in Northern Greece” identified three distinct clusters among OSAS patients, each characterized by varying severity of the condition, obesity levels, and comorbidities. The first cluster included patients with moderate OSAS, obesity, and high Epworth Sleepiness Scale (ESS) scores but without significant comorbidities. The second cluster consisted of patients with severe OSAS, severe obesity, multiple comorbidities, and the highest ESS scores. The third cluster comprised patients with severe OSAS and obesity, high ESS scores, but without comorbidities. These findings underscore the heterogeneity of OSAS presentations in primary healthcare settings and highlight the importance of personalized treatment approaches. By recognizing specific phenotypes, clinicians can tailor interventions more effectively, addressing not only the severity of OSAS but also associated comorbid conditions and patient-reported outcomes like daytime sleepiness [18].

Syed F. Hussain et al. (2004) conducted a prospective, randomized, single-blind crossover trial comparing four weeks of auto-titrating CPAP (APAP) versus fixed-pressure CPAP in ten CPAP-naive patients with moderate to severe OSA. Both interventions achieved equivalent improvements in symptoms and AHI, with similar side-effect profiles and compliance. Interestingly, despite expectations of greater comfort, a majority of participants (6 out of 10) preferred fixed CPAP over APAP. These results are in concordance with our paper and suggest that APAP does not necessarily confer superior tolerability or user satisfaction in this population [19].

The National Guidelines of Sleep Breathing Disorders in Adults recommend choosing CPAP over APAP in patients with significant heart disease [4]. This idea was furthered by Karasulu L et al., who investigated cardiovascular autonomic outcomes by comparing heart-rate variability (HRV) in patients undergoing APAP versus conventional CPAP. In this single-center study, conventional CPAP yielded significantly greater improvements in HRV metrics during Stage 2 sleep than APAP, suggesting enhanced parasympathetic activation and more stable cardiovascular control with constant-pressure therapy. These findings underscore potential autonomic advantages of fixed-pressure delivery, with implications for cardiovascular risk reduction in OSA management [20]. Our results suggest that atrial fibrillation may be a factor in non-compliance or non-adherence to PAP therapy. CPAP should be used in patients who are refractory to APAP treatment, especially those with higher heart-rate variability, such as atrial fibrillation.

More patient characteristics and comorbidities should be considered in future studies to better understand PAP device choice. A recent study from 2023 by Bironneau et al. [21] evaluated fixed CPAP versus APAP over a three-month period in 801 patients with severe OSA. The results showed similar efficacy in AHI reduction and symptom alleviation between modalities, with no meaningful difference in adherence or patient outcomes [21]. This study adds robust, contemporary evidence affirming clinical equivalence between CPAP modalities in severe OSA, but it did not consider comorbidities or certain demographic factors.

Similar to our paper, a few studies in the literature used usage time as a marker to evaluate the OSA patient under PAP therapy [22,23,24]. The low utilization times indicated poorer adherence due to poor comfort, which caused the patient to stop using the device for the rest of the night. Ip S. et al. (2012) conducted a meta-analysis of 24 randomized trials comparing auto-titrating and fixed CPAP therapy. APAP delivered modest but statistically significant benefits, improving daily usage by approximately 11 min and lowering ESS scores by 0.5 points. Conversely, fixed CPAP achieved slightly better minimum oxygen saturation. Overall, both modalities demonstrated comparable outcomes in AHI reduction, sleep quality, and quality of life, suggesting the acceptability of either approach depending on patient needs [23].

Fuchs et al. examined microarousal frequency related to pressure swings during APAP therapy in a cohort of patients with moderate to severe OSA. Using polysomnography, the study reported low overall microarousal rates, though slight increases were noted specifically during pressure increases [25]. These findings highlight the potential for pressure variability in APAP devices to induce subtle sleep fragmentation [26], emphasizing the importance of considering sleep architecture effects when choosing a therapy modality. This could explain the lower compliance we observed in patients undergoing APAP treatment with high pressure variability (>10 cm H_2_O).

A potential resolution to the problem of pressure variability in patients under Auto-PAP treatment could be explained by Alves and colleagues. Their study evaluated the clinical and economic outcomes of transitioning patients with well-controlled OSA from auto-titrating PAP (APAP) to fixed-pressure CPAP. Among 93 participants, both modalities achieved equally effective correction of obstructive events and improvement in daytime somnolence, with no significant differences in adherence or device tolerability. Notably, selecting fixed CPAP pressures based on the 90th or 95th percentile values from APAP performance proved a reliable alternative to in-laboratory titration. Furthermore, this transition strategy yielded estimated cost savings exceeding EUR 10,000 over two years. These findings suggest that, for patients already well-adapted to APAP, transitioning to fixed CPAP is a clinically viable and economically advantageous approach [27].

Similarly, Testelmans et al. conducted a retrospective study directly comparing auto-titrating PAP and in-laboratory titrated fixed-pressure CPAP in over 100patients with moderate to severe OSA. Although no significant differences were observed between the groups in terms of residual AHI as assessed by polysomnography, patients in the fixed-CPAP titration group were titrated to significantly lower pressure levels and exhibited significantly lower average leak rates compared to those titrated with APAP [28]. Another study of 269 patients found no significant leak-related differences between CPAP and APAP, but suggested that smoking, high pressure, and oronasal masks are associated with above-median leaks [29].

In a randomized, single-blind crossover trial involving 20 patients with moderate to severe OSA, Galetke and colleagues demonstrated that both fixed CPAP and APAP significantly reduced AHI and improved daytime sleepiness without clinically relevant differences between modalities. Leak rates and nightly usage were also comparable across treatment arms, though a trend toward fewer leaks was observed with APAP. The overall APAP variability remained relatively modest. Importantly, approximately two-thirds of participants expressed a preference for the automatic mode. The findings indicate that while both CPAP and APAP provide equivalent therapeutic efficacy and adherence, the reduced leak tendency and higher patient preference for APAP may support its selection by both the patient and the specialist [30].

### Limitations

This study has several limitations that should be considered when interpreting the findings.

First, as a retrospective analysis based on computerized system entries, the accuracy and completeness of the data depended on the records entered by other physicians and the utilization reports provided by PAP device suppliers. The study team had no control over potential inconsistencies or missing information in the dataset, which may have affected the reliability of the results. A significant limitation is the missing data for the adherence metric (average hours of use) in approximately 41% of the cohort. This was strictly due to inconsistencies in manual data entry into the hospital system. While this reduces the statistical power of the adherence analysis, the demographic and clinical profiles of the subsample (n = 211) were similar to the total cohort, suggesting limited bias. Furthermore, adherence data relies on device-recorded usage time at therapeutic pressure. While this is the standard metric for reimbursement in Romania, it does not objectively verify sleep via EEG, although utilizing PAP devices while awake is generally poorly tolerated by patients.

Second, the relatively small sample size limits the statistical power of the study. While the findings provide valuable insights into PAP adherence/compliance patterns, a larger cohort would enhance the generalizability of the conclusions. Additionally, some key variables, such as patient-reported side effects, mask-fit issues, and subjective sleep quality, were not assessed, which could have provided a more comprehensive understanding of adherence challenges. Additionally, data regarding mask leaks were not consistently available in the hospital database. We acknowledge that high-pressure variability can sometimes be an artifact of high air leaks rather than physiological obstruction. However, regardless of the cause (leak vs. respiratory event), large pressure swings represent a disruptive factor for sleep quality and likely contribute to lower compliance.

Third, the cohort included only patients who met the initial eligibility criteria for reimbursement, introducing a selection bias toward higher adherence. Consequently, our findings are specific to the population actively engaged in the national compensation program and may not be generalizable to patients who fail to initiate therapy or purchase devices out-of-pocket. However, identifying risk factors for non-adherence in this pre-selected, motivated group (such as high-pressure variability) highlights pertinent barriers that persist even among compliant users. It also implies that the patients included have relatively similar parameters, without significant differences, which resulted in a lack of polarization of the results. These notions may indicate that the results are suggestive rather than conclusive.

Regarding the statistical models, we acknowledge that the predictive accuracy of the multivariate analyses was modest (Nagelkerke R^2^ of ~21–23%). This suggests that adherence and compliance are multifactorial behaviors influenced by variables not captured in our dataset, such as psychological factors, social support, or mask comfort. Furthermore, while specific variables like arterial hypertension showed significance, the overall compliance model did not reach statistical significance, warranting caution in interpretation.

Finally, this study did not account for external factors that may influence CPAP adherence or compliance, such as socioeconomic status, psychological factors, education level, living and housing situation, or long-term patient follow-up beyond the study period. Literature data on Auto-PAP variability are limited, and even more so regarding the relationship between pressure variability and adherence/compliance scores. Future research should be carried out considering these aspects to develop a more integrated approach to improving PAP compliance.

## 5. Conclusions

This study highlights the critical factors influencing CPAP adherence and compliance among patients with obstructive sleep apnea (OSA). While CPAP therapy has proven effective in improving sleep quality and reducing OSA-related symptoms, adherence and compliance remain significant challenges, particularly among patients with high-pressure variability in Auto-PAP devices and those with comorbid conditions such as COPD or atrial fibrillation. The findings underscore the importance of personalized patient education, regular follow-ups, and proper device titration to improve long-term adherence. Additionally, the role of healthcare providers, including the emerging concept of “sleep nurses,” is pivotal in addressing barriers to compliance, such as mask discomfort and side effects. Geographic disparities in adherence, with lower compliance in rural areas, further emphasize the need for accessible healthcare services and patient support systems. By identifying clinical markers that may impair compliance and adherence, this study calls for targeted interventions to improve outcomes. Future research should focus on standardizing data collection and exploring the impact of patient education and support systems on adherence, ultimately reducing the economic and health burdens associated with untreated OSA.

## Figures and Tables

**Table 1:** Comparison of demographic data.

Compliance	Overall, N = 359	Poor, N = 26	Good, N = 333	*p*-Value ^1^
**Sex, n (%)**				0.98
Male	220 (61)	16 (62)	204 (61)	
Female	139 (39)	10 (38)	129 (39)	
**Age, median (IQR)**	60 (53–69)	59 (53–61)	61 (53–69)	0.094
**Environment, n (%)**				0.34
Rural	71 (21)	7 (28)	64 (20)	
Urban	274 (79)	18 (72)	256 (80)	
**Smoking, n (%)**				0.35
Ex-Smoker	126 (36)	10 (40)	116 (36)	
Smoker	68 (20)	7 (28)	61 (19)	
Non-Smoker	152 (44)	8 (32)	144 (45)	

^1^ Pearson’s Chi-squared test; Wilcoxon rank-sum test; Fisher’s exact test.

**Table 2:** Comorbidities of the studied group.

Characteristic	Overall, N = 359	Poor, N = 26	Good, N = 333	*p*-Value ^1^
**Body Mass Index, median (IQR)**	34 (30–38)	34 (30–39)	34 (30–38)	0.88
**Obesity status, n (%)**				0.87
Non-Obese	79 (23)	6 (24)	73 (23)	
Obese	269 (77)	19 (76)	250 (77)	
**Nasal septum deviation, n (%)**				0.049
Absent	271 (77)	24 (92)	247 (75)	
Present	83 (23)	2 (7.7)	81 (25)	
**Nasal polyposis, n (%)**				>0.99
Absent	335 (95)	25 (96)	310 (95)	
Present	19 (5.4)	1 (3.8)	18 (5.5)	
**Arterial hypertension, n (%)**				0.025
Absent	96 (27)	12 (46)	84 (26)	
Present	256 (73)	14 (54)	242 (74)	
**Atrial fibrillation, n (%)**				0.59
Absent	290 (82)	23 (88)	267 (82)	
Present	63 (18)	3 (12)	60 (18)	
**Heart failure, n (%)**				0.58
Absent	247 (70)	17 (65)	230 (71)	
Present	105 (30)	9 (35)	96 (29)	
**Diabetes, n (%)**				0.63
Absent	245 (70)	17 (65)	228 (70)	
Present	107 (30)	9 (35)	98 (30)	
**COPD, n (%)**				0.27
Absent	297 (84)	20 (77)	277 (85)	
Present	55 (16)	6 (23)	49 (15)	
**Asthma, n (%)**				0.71
Absent	323 (92)	25 (96)	298 (91)	
Present	29 (8.2)	1 (3.8)	28 (8.6)	

^1^ Fisher’s exact test; Wilcoxon rank-sum test; Pearson’s Chi-squared test.

**Table 3:** Comparison of nocturnal cardiorespiratory polygraphy, type of device used, and type of mask.

Characteristic	Overall, N = 359	Poor, N = 26	Good, N = 333	*p*-Value ^1^
Apnea-hypopnea index, median (IQR)	40 (28–60)	40 (30–57)	40 (28–60)	0.95
Oxygen desaturation index, median (IQR)	46 (29–64)	50 (35–63)	45 (28–64)	0.55
Times spent with SaO_2_ < 90% during polygraphy, median (IQR)	25 (10–46)	42 (15–77)	24 (9–45)	0.13
Minimum SaO_2_ during polugraphy, median (IQR)	71 (60–80)	73 (66–80)	71 (59–80)	0.33
Severity, n (%)				0.86
Mild	12 (3.5)	0 (0)	12 (3.8)	
Moderate	83 (24)	7 (27)	76 (24)	
Severe	248 (72)	19 (73)	229 (72)	
Type of home device (CPAP/APAP/BPAP), n (%)				0.13
APAP	226 (64)	21 (81)	205 (63)	
BIPAP	53 (15)	1 (3.8)	52 (16)	
CPAP	74 (21)	4 (15)	70 (21)	
MASK, n (%)				0.030
Oronasal	185 (71)	10 (50)	175 (73)	
Nasal	75 (29)	10 (50)	65 (27)	

^1^ Wilcoxon rank-sum test; Fisher’s exact test; Pearson’s Chi-squared test.

**Table 4:** Comparison of CPAP pressure, Auto-PAP variability (CPAPmax − CPAPmin), and BiPAP pressure support.

Characteristic	Overall, N = 359	Poor, N = 26	Good, N = 333	*p*-Value ^1^
CPAP pressure, median (IQR)	11.00 (9.00–12.00)	11.50 (10.00–12.50)	11.00 (9.00–12.00)	0.78
CPAPmax PRESSURE − CPAPmin PRESSURE (Variability in Auto-CPAP pressure), median (IQR)	10.0 (6.3–16.0)	16.0 (12.0–16.0)	10.0 (6.0–16.0)	0.001
BPAP pressure support, n (%)	5.00 (4.00–8.00)	5.00 (5.00–5.00)	5.00 (4.00–8.00)	>0.99

^1^ Wilcoxon rank-sum test.

**Table 5:** Demographic data in relation to the adherence groups.

Characteristic	Overall, N = 211	Poor, N = 109	Good, N = 102	*p*-Value ^1^
Sex, n (%)				0.93
Male	133 (63)	69 (63)	64 (63)	
Female	78 (37)	40 (37)	38 (37)	
Age, median (IQR)	62 (54–70)	62 (54–69)	61 (54–70)	0.55
Environment, n (%)				0.24
Rural	31 (15)	19 (18)	12 (12)	
Urban	171 (85)	85 (82)	86 (88)	
Smoking, n (%)				0.95
Ex-Smoker	80 (39)	40 (38)	40 (40)	
Smoker	30 (15)	16 (15)	14 (14)	
Non-Smoker	96 (47)	49 (47)	47 (47)	

^1^ Pearson’s Chi-squared test; Wilcoxon rank-sum test.

**Table 6:** Comorbidities in relation to the adherence groups.

Characteristic	Overall, N = 211	Poor, N = 109	Good, N = 102	*p*-Value ^1^
Body Mass Index, median (IQR)	33 (30–38)	34 (31–38)	32 (30–37)	0.13
Obesity status, n (%)				0.090
Non-Obese	42 (20)	17 (16)	25 (25)	
Obese	167 (80)	92 (84)	75 (75)	
Nasal septum deviation, n (%)				0.023
Absent	153 (73)	86 (80)	67 (66)	
Present	57 (27)	22 (20)	35 (34)	
Nasal polyposis, n (%)				0.10
Absent	199 (95)	105 (97)	94 (92)	
Present	11 (5.2)	3 (2.8)	8 (7.8)	
Arterial hypertension, n (%)				0.30
Absent	51 (24)	23 (21)	28 (27)	
Present	159 (76)	85 (79)	74 (73)	
Atrial fibrillation, n (%)				0.018
Absent	167 (80)	79 (73)	88 (86)	
Present	43 (20)	29 (27)	14 (14)	
Heart Failure, n (%)				0.42
Absent	142 (68)	70 (65)	72 (71)	
Present	67 (32)	37 (35)	30 (29)	
Diabetes, n (%)				0.87
Absent	150 (72)	77 (71)	73 (72)	
Present	59 (28)	31 (29)	28 (28)	
COPD, n (%)				0.25
Absent	179 (85)	95 (88)	84 (82)	
Present	31 (15)	13 (12)	18 (18)	
Asthma, n (%)				0.27
Absent	192 (91)	101 (94)	91 (89)	
Present	18 (8.6)	7 (6.5)	11 (11)	

^1^ Pearson’s Chi-squared test; Wilcoxon rank-sum test.

**Table 7:** Comparison of nocturnal cardiorespiratory polygraphy, type of device used, and type of mask in relation to the adherence groups.

Characteristic	Overall, N = 211	Poor, N = 109	Good, N = 102	*p*-Value ^1^
Apnea/Hypopnea Index, Median (IQR)	42 (30–59)	45 (30–60)	39 (28–57)	0.18
Oxygen Desaturation Index, Median (IQR)	47 (29–65)	52 (33–70)	44 (27–62)	0.026
Times spent with SaO_2_ < 90% during polygraphy, Median (IQR)	24 (12–40)	21 (12–35)	31 (7–48)	0.48
Minimum SaO_2_ during polugraphy, Median (IQR)	71 (61–80)	71 (60–79)	70 (64–81)	0.54
SEVERITY, n (%)				0.35
Mild	7 (3.5)	2 (1.9)	5 (5.1)	
Moderate	45 (22)	21 (20)	24 (24)	
Severe	150 (74)	80 (78)	70 (71)	
Type of home device (CPAP/APAP/BPAP), n (%)				0.027
APAP	137 (65)	80 (73)	57 (56)	
BIPA	29 (14)	12 (11)	17 (17)	
CPAP	45 (21)	17 (16)	28 (27)	
MASK, n (%)				0.59
Oronasal	125 (76)	58 (74)	67 (78)	
Nasal	39 (24)	20 (26)	19 (22)	

^1^ Wilcoxon rank-sum test; Fisher’s exact test; Pearson’s Chi-squared test.

**Table 8:** Comparison of CPAP pressure, Auto-PAP variability (CPAPmax − CPAPmin), and BiPAP pressure support in relation to the adherence groups.

Characteristic	Overall, N = 211	Poor, N = 109	Good, N = 102	*p*-Value ^1^
CPAP pressure, median (IQR)	11.00 (10.00–12.00)	11.00 (10.00–12.00)	11.00 (9.75–12.00)	0.98
CPAPmax PRESSURE − CPAPmin PRESSURE (variability in Auto-CPAP pressure), median (IQR)	10.0 (6.0–13.3)	10.0 (6.0–12.3)	10.0 (6.0–16.0)	0.83
BPAP pressure support, median (IQR)	5.00 (4.00–8.00)	4.00 (4.00–5.75)	5.00 (4.00–8.00)	0.18

^1^ Wilcoxon rank-sum test.

**Table 9:** Multivariate analysis for compliance.

	B	S.E.	Wald	df	Sig.	Exp(B)	95% C.I. for EXP(B)	
							Lower	Upper
Age	0.054	0.036	2.350	1	0.125	1.056	0.985	1.132
Sex(Male)	0.481	0.655	0.539	1	0.463	1.618	0.448	5.844
Non-Smoker			1.530	2	0.465			
Ex-Smoker	−0.814	0.678	1.439	1	0.230	0.443	0.117	1.675
Smoker	−0.642	0.755	0.724	1	0.395	0.526	0.120	2.309
Obesity status (present)	−1.027	0.766	1.797	1	0.180	0.358	0.080	1.607
Nasal septum deviation (present)	1.562	0.870	3.224	1	0.073	4.769	0.867	26.244
Nasal polyposis (present)	17.563	11,220.338	0.000	1	0.999	42,397,225.364	0.000	
Arterial hypertension (present)	1.655	0.693	5.701	1	**0.017**	**5.233**	**1.345**	**20.357**
Atrial fibrillation (present)	−0.273	0.795	0.118	1	0.732	0.761	0.160	3.616
Heart failure (present)	−0.969	0.719	1.819	1	0.177	0.379	0.093	1.551
Diabetes (present)	0.074	0.675	0.012	1	0.912	1.077	0.287	4.046
COPD (present)	−1.382	0.860	2.585	1	0.108	0.251	0.047	1.354
Asthma (present)	0.702	1.238	0.322	1	0.571	2.019	0.178	22.863
Type of home device (BiPAP)			2.613	2	0.271			
Type of home device (CPAP)	2.159	1.373	2.473	1	0.116	8.666	0.587	127.833
Type of home device (APAP)	−0.092	0.716	0.016	1	0.898	0.912	0.224	3.716
Type of mask used (Oronasal)	0.855	0.629	1.848	1	0.174	2.350	0.686	8.057
Constant	−1.430	2.238	0.408	1	0.523	0.239		

**Table 10:** Multivariate analysis for adherence.

	B	S.E.	Wald	df	*p*-Value	Odds Ratio	95% C.I. for EXP(B)	
							Lower	Upper
Age	0.015	0.019	0.650	1	0.420	1.015	0.979	1.053
Sex (Male)	−0.387	0.418	0.856	1	0.355	0.679	0.299	1.542
Non-Smoker			0.666	2	0.717			
Ex-Smoker	0.331	0.468	0.501	1	0.479	1.393	0.557	3.484
Smoker	−0.055	0.565	0.009	1	0.923	0.947	0.313	2.864
Obesity status (present)	−0.884	0.488	3.288	1	0.070	0.413	0.159	1.074
Nasal septum deviation (present)	0.921	0.447	4.257	1	**0** **.039**	**2.513**	**1.047**	**6.029**
Nasal polyposis (present)	0.290	0.802	0.131	1	0.717	1.337	0.278	6.433
Arterial hypertension (present)	−0.437	0.468	0.871	1	0.351	0.646	0.258	1.617
Atrial fibrillation (present)	−1.405	0.523	7.208	1	**0** **.007**	**0** **.245**	**0** **.088**	**0** **.684**
Heart failure (present)	−0.336	0.431	0.607	1	0.436	0.715	0.307	1.664
Diabetes (present)	0.053	0.443	0.014	1	0.904	1.055	0.443	2.513
COPD (present)	0.912	0.626	2.123	1	0.145	2.489	0.730	8.488
Asthma (present)	−0.164	0.666	0.061	1	0.805	0.848	0.230	3.132
Type of home device (BiPAP)			5.276	2	0.071			
Type of home device (CPAP)	0.639	0.583	1.201	1	0.273	1.894	0.604	5.941
Type of home device (APAP)	1.039	0.468	4.925	1	**0** **.026**	**2.826**	**1.129**	**7.071**
Type of mask used (Oronasal)	−0.135	0.450	0.089	1	0.765	0.874	0.362	2.113
Constant	0.196	1.344	0.021	1	0.884	1.217		

## Data Availability

The raw data supporting the conclusions of this article will be made available by the authors on request. The data are not publicly available due to ethical and national law restrictions.

## Abbreviations

The following abbreviations are used in this manuscript:

OSA/OSAS	Obstructive Sleep Apnea/Obstructive Sleep Apnea Syndrome
CPAP	Continuous Positive Airway Pressure
Auto-PAP/APAP	Automated Positive Airway Pressure
AHI	Apnea-Hypopnea Index
NIV	Non-Invasive Ventilation
CNAS	Casa Nationala de Asigurari de Sanatate (National Health Insurance House)
BMI	Body Mass Index
COPD	Chronic Obstructive Pulmonary Disease
ΔP (Delta P)	Pressure Support
PSQI	Pittsburgh Sleep Quality Index
